# Local genic base composition impacts protein production and cellular fitness

**DOI:** 10.7717/peerj.4286

**Published:** 2018-01-16

**Authors:** Erik M. Quandt, Charles C. Traverse, Howard Ochman

**Affiliations:** Department of Integrative Biology, University of Texas at Austin, Austin, TX, United States of America

**Keywords:** G + C content, Base composition, Translation rate, Evolution, Codon usage

## Abstract

The maintenance of a G + C content that is higher than the mutational input to a genome provides support for the view that selection serves to increase G + C contents in bacteria. Recent experimental evidence from *Escherichia coli* demonstrated that selection for increasing G + C content operates at the level of translation, but the precise mechanism by which this occurs is unknown. To determine the substrate of selection, we asked whether selection on G + C content acts across all sites within a gene or is confined to particular genic regions or nucleotide positions. We systematically altered the G + C contents of the GFP gene and assayed its effects on the fitness of strains harboring each variant. Fitness differences were attributable to the base compositional variation in the terminal portion of the gene, suggesting a connection to the folding of a specific protein feature. Variants containing sequence features that are thought to result in rapid translation, such as low G + C content and high levels of codon adaptation, displayed highly reduced growth rates. Taken together, our results show that purifying selection acting against A and T mutations most likely results from their tendency to increase the rate of translation, which can perturb the dynamics of protein folding.

## Introduction

Bacterial species exhibit a wide range of genomic base compositions, ranging from 13% to 75% G + C (Thomas et al., 2008; McCutcheon & Moran, 2010). Because mutations in bacteria are universally biased towards A and T, the maintenance of a G + C content that is higher than the mutational input to a genome implies a role of natural selection in shaping genomic base composition (Hershberg & Petrov, 2010; Hildebrand, Meyer & Eyre-Walker, 2010). However, it has been difficult to establish the specific traits or circumstances for which higher G + C contents might be advantageous. Many hypotheses have been suggested to explain the differences in genomic G + C contents among organisms, most commonly citing an association and advantage of a particular base composition with an environmental variable (Singer & Ames, 1970; Kagawa et al., 1984; McEwan, Gatherer & McEwan, 1998; Foerstner et al., 2005; Rocha & Feil, 2010). However, these correlations are rarely robust across taxa (Hurst & Merchant, 2001) or are complicated by other factors (Rocha & Feil, 2010) leaving the actual basis of the variation unknown. Moreover, the selection coefficients necessary to favor individual nucleotide substitutions that alter genomic base composition seem unrealistically small, even given the large effective population sizes of bacteria.

Prior research on *E. coli* has demonstrated that strains expressing genes of lower G + C contents had slower doubling times, even when the sequences of the encoded proteins were identical (Raghavan, Kelkar & Ochman, 2012). Furthermore, this GC-effect was dependent on translation such that isogenic constructs lacking ribosome binding sites did not recapitulate the fitness defect (Raghavan, Kelkar & Ochman, 2012). These findings suggest that selection acts at the level of individual genes and not on genomic base composition as a whole, and that the translational process is somehow affected by changes in base composition.

Shifting the focus towards the translation of individual genes mitigates some of the concerns about the efficacy of selection needed to favor compositional changes at individual sites but raises questions about how cellular fitness could be linked to the G + C content of a gene. One potential explanation is that genes of different base composition differ in their patterns of ribosome turnover such that ribosomes become limiting during rapid growth (Andersson & Kurland, 1990; Plotkin & Kudla, 2011; Weiße et al., 2015; Gorochowski et al., 2016). Selection for translational efficiency is reflected in the sequences of highly expressed genes, which are biased towards using codons that can be translated rapidly due to high concentrations of their cognate tRNAs (Ikemura, 1985; Andersson & Kurland, 1990; Dong, Nilsson & Kurland, 1996). It is possible that differences in the speed at which A/T or G/C bases are translated, or the tendency to form sequence motifs that affect decoding, result in a similar form of selective pressure on base composition. Alternatively, differences in base composition might alter the rate of translation and affect the folding of nascent polypeptides. An inappropriate rate of translation could cause proteins to misfold and aggregate, and be detrimental to the cell.

To distinguish the specific substrate of G + C selection, we asked if selection for G + C content is acting over the entire gene or is confined to certain motifs or nucleotide positions. To accomplish this, we synthesized a large set of gene constructs comprising variants of mosaic base compositions and tested for any growth defects associated with each variant when expressed under a variety of promoters. We found that the fitness benefit incurred from increased G + C contents varied with genic location and was associated with a reduced rate of co-translational protein folding. Based on these findings, selection for higher G + C contents serves to counter the increased rate of translation caused by A and T mutations, which disrupt protein folding dynamics by decreasing the stability of mRNA secondary structures.

## Materials and Methods

### Bacterial strains and growth conditions

Growth and fluorescence assays were performed with *E. coli* strain BW25113 (*F*-, Δ*(araD-araB)567*, Δ*lacZ4787*(::rrnB-3), *λ*^−^, *rph-1*, Δ*(rhaD-rhaB)568*, *hsdR514*) (Datsenko & Wanner, 2000; Baba et al., 2006). Cells were grown in Lysogeny Broth (LB) of the Lennox variety (5 g/L NaCl). When appropriate, antibiotics were supplemented at the following concentrations: ampicillin (Amp) (100 µg/mL), kanamycin (Kan) (30 µg/mL), streptomycin (Strep/Sm) (100 µg/mL).

### Design of recoded GFP-L, M and H genes

The 239 amino-acid superfolder GFP (sfGFP) protein-coding sequence served as the basis for GFP designs. This sequence has been shown to be a superior reporter for gene expression due to its fast folding kinetics and improved stability (Pédelacq et al., 2006). The sfGFP DNA sequence was recoded at synonymous sites using the EuGene genetic optimization software (Gaspar et al., 2012). To create the GFP-L, M, and H variants, the sfGFP gene was recoded three times, with settings in EuGene set to target G + C contents of 40%, 50%, and 60% and with the CAI of each variant optimized to the *E. coli* MG1655 genome. The first 51 base pairs (52.9% G + C) and last 54 base pairs (48.1% G + C) were held constant in all gene variants as these regions are known to alter gene expression (Grunberg-Manago, 1999; Kudla et al., 2009; Goodman, Church & Kosuri, 2013; Umu et al., 2016). The CA- and GT-enriched variants of the GFP-L terminal fragment were recoded manually using EuGene by changing each codon to a CA- or GT-rich version wherever possible. Sequences of all gene variants used in this study are presented in Table S2.

### CAI and mRNA folding energy calculations

Codon adaptation index (CAI) (Sharp & Li, 1987) values for each GFP variant were calculated using the default options of the ‘cai’ function in Emboss 6.6.0 (Rice, Longden & Bleasby, 2000) based on a set of 40 highly expressed genes that included 37 ribosomal proteins and 3 elongation factors (Sharp et al., 2005; Hilterbrand, Saelens & Putonti, 2012). The folding energy of each GFP variant was calculated using RNALfold (Hofacker, Priwitzer & Stadler, 2004) with default options. These values are presented in Table S1.

### Gene and plasmid construction

The GFP-L, M, and H gene sequences were each subdivided into three non-overlapping, roughly equal-sized sequence fragments: 5′-distal, proximal, and terminal. The 5′-distal fragment spanned base pair positions 1–255 (encoding amino acids 1–68), the proximal fragment spanned base pair positions 256–459 (encoding amino acids 69–153), and the terminal-fragment spanned base pair positions 460–717 (encoding amino acids 154–239). These nine sequence fragments were synthesized as dsDNA gBLOCK gene fragments (IDT).

To create the full-length GFP-L, M and H genes, as well as the compositional mosaics of high, medium and low-GC fragments, the selected fragments were assembled using either the Ligase Cycling Reaction (LCR) method (De Kok et al., 2014) or a modified Gibson assembly method (Paetzold et al., 2013), which employed bridging oligos that overlapped with ≈30 bp of each fragment to mediate assembly. Full-length genes were assembled into pUC19 and then subcloned by Gibson assembly (Gibson et al., 2009) into the pFAB-series expression vectors—pFAB3701, pFAB3845, pFAB3857, pFAB3833, pFAB3665 and pFAB3689 (Mutalik et al., 2013)—in place of the previously encoded RFP gene. The pFAB-series expression vectors contain well-characterized constitutive promoters of varying strength as well as a bicistronic design that has been shown to normalize translation initiation strength regardless of the sequence in the translation initiation region of the expressed gene. By using these vectors, we aimed to eliminate fluctuations in expression due to differential efficiencies of translation initiation caused by the coding sequence and to instead focus on effects arising from the elongation phase. Gene and promoter sequences of all constructs were verified by Sanger sequencing.

### Cell growth and fluorescence measurements

Strains were grown overnight at 37 °C with shaking at 200 rpm in LB supplemented with the appropriate antibiotic for plasmid maintenance. Overnight cultures were used to inoculate assay media at a starting OD_600_ = 0.05. 100 µL of the inoculated assay media were added to a well of a 96-well flat-bottom plate (Corning, Inc., Corning, NY, USA), and plates were incubated at 37 °C with shaking at 600 rpm. OD_600_ readings were taken on a VICTOR X3 (Perkin Elmer) plate reader. Growth rates were calculated using the Gompertz equation for bacterial cell growth (Zwietering et al., 1990) using GraphPad Prism Version 6.0. For fluorescence measurements, 10 µL of culture media were added to 90 µL of ddH_2_0 in a black, clear bottom 96-well plate (Corning, Inc., Corning, NY, USA) and assayed at an excitation/emission wavelength of 485/535 nm in the plate reader. Simultaneously, an OD_600_ measurement was taken, and fluorescence was calculated as RFU_485/535_/OD_600_.

### Strain construction

The P90Q mutation was introduced into the *rpsL* gene of *E. coli* strain BW25113 using the oligo-mediated *λ* red ‘recombineering’ method (Ellis et al., 2001). Briefly, strain BW25113 was transformed with plasmid pKD46 (Datsenko & Wanner, 2000), which contains the *λ* red recombinase genes under the control of the AraC arabinose-inducible promoter. Cells were grown in LB to an OD_600_ ≈ 0.4, then treated with 0.1% L-Arabinose for 1 hr to induce expression of the *λ* red recombinase genes. Cells were made electrocompetent and then electroporated with 1 µg of oligo EQ_rpsL_P90Q (A*A*GCG CAC CAC GTA CGG TGT GGT AAC GAA CAC CC TGG AGG TCT TTA ACA CGA CCG CCA CGG ATC AGG A*T*C), allowed to recover, and plated on LB-strep agar. The *rpsL* gene of the resulting Sm^R^ strain was PCR amplified and sequenced to confirm the introduction of the P90Q mutation.

## Results

The base composition of the GFP gene was systematically modified from the whole-gene to the individual-nucleotide levels to pinpoint the specific features responsible for altering *E. coli* growth rates. We initially assayed the fitness effects and expression levels of GFP genes recoded across synonymous sites to span a range of G + C contents. We then synthesized GFP genes that were mosaics of high- and low-GC fragments to localize the source of fitness defects to a particular region of the gene. This guided our focus towards specific codon positions at which synonymous rare codon substitutions were capable of restoring fitness to wild-type levels.

### Overall base composition of GFP genes modulates cellular fitness and gene expression levels

To test the effects of genic G + C content on cellular fitness, we recoded the nucleotide sequence of the GFP gene to produce coding sequences with a consistently low (L; 41%), medium (M; 50%), or high (H; 59%) G + C content across the entire gene (Table S2). This was achieved by recoding genes with synonymous codons that differ in G + C content, which ensured that the protein sequence encoded by the various gene constructs remained identical.

**Figure 1:**
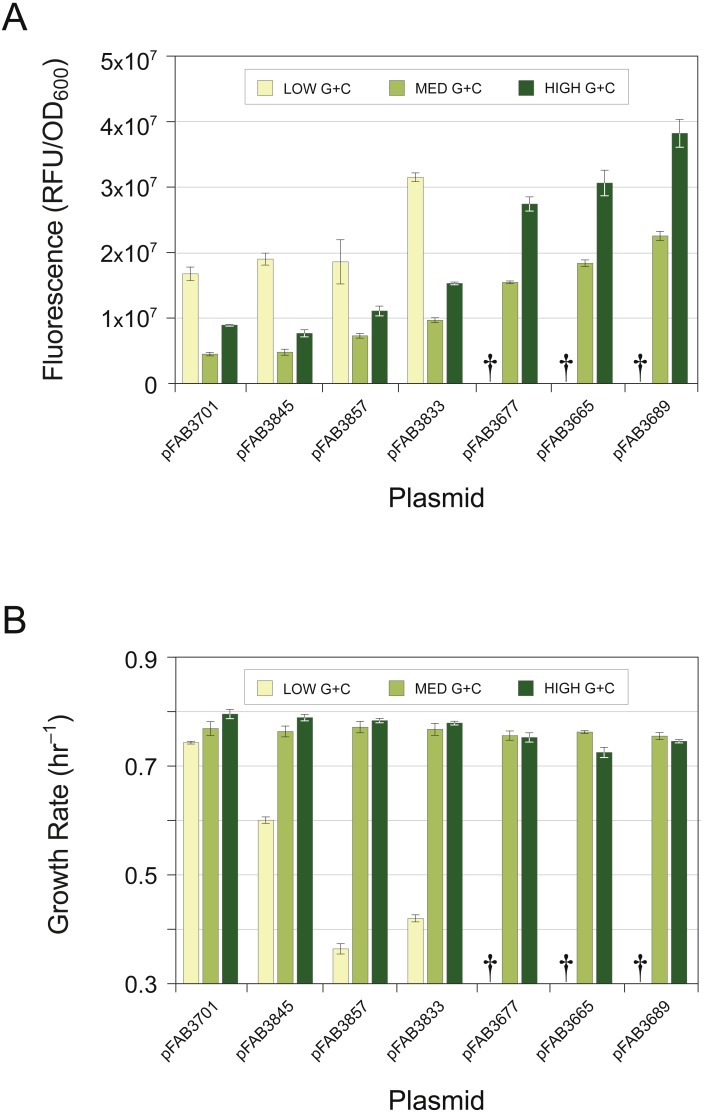
Effects of promoter strength and base composition on expression level and cellular growth rate. GFP genes were recoded at synonymous sites to possess a consistently low (yellow; 41%), medium (light green; 50%) or high (dark green; 59%) G + C content over the entire gene. (A) GFP constructs of different G + C contents tested for expression levels in multiple pFAB vectors, displayed in order of increasing promoter strength. Expression levels determined by intensity of cell fluorescence. Daggers (†) denote constructs refractory to cloning, all of which were of low G + C contents. (B) Fitness measurements of strains expressing GFP constructs from (A) determined by exponential-phase growth rates. Daggers (†) denote constructs not assayed. Bars represent the mean ± standard deviation of three biological replicates.

The three GFP genes, GFP-L, GFP-M, and GFP-H, were each tested for expression and growth-rate characteristics in a series of pFAB vectors containing promoters of different strengths (Mutalik et al., 2013). Overall levels of expression, as measured by cellular GFP fluorescence, depended on the intrinsic strength of the pFAB promoter; however, in each of the expression vectors in which all three GFP constructs could be cloned, we observed the identical trend: the GFP gene of low G + C contents (GFP-L) expressed at higher levels than the gene of high G + C contents (GFP-H), and the gene of intermediate G + C contents (GFP-M) expressed at the lowest level (Fig. 1A).

Of particular note is that the GFP-L gene, which typically expresses at the highest level, could not be stably cloned into the three expression vectors possessing the strongest promoters (Fig. 1A). Transformations of these constructs yielded dramatically reduced numbers of recombinant clones and were usually not fluorescent (but see later section). Those few clones that were recovered were found to possess mutations in the coding or promoter sequences, suggesting that high-level expression of the GFP-L gene is lethal, leading to selection for mutations that alleviate its toxicity.

The toxicity of the GFP-L gene was similarly apparent from the growth rates of strains that were capable of expressing this gene from weaker promoters: growth rates of strains expressing the GFP-L gene decreased sharply with increasing expression level (Fig. 1B). In contrast, expression of the GFP-M or GFP-H genes did not adversely affect growth rates, even at the highest levels of expression. These results agree with the previous findings that recoded GFP genes of low G + C contents impose a fitness cost when expressed in *E. coli* (Raghavan, Kelkar & Ochman, 2012; Kelkar, Phillips & Ochman, 2015) and show that this fitness decrement is dose-dependent with protein expression levels (Fig. S1).

### Compositional mosaics reveal sequence features underlying cellular toxicity

To determine whether a specific sequence motif or the G + C content of a particular genic region was responsible for the observed differences in gene expression and cellular toxicity, we shuffled portions of the previously synthesized GFP genes to produce compositional mosaics. Each of the three compositional variants of the GFP from above was subdivided into three gene fragments representing the 5′-distal, proximal, and terminal regions. These fragments were reassembled to produce mosaic genes corresponding to all 27 possible full-length sequence combinations. The compositional mosaics were assayed in the pFAB3857 vector, which was selected on account of its intermediate level of expression (Fig. 1A) and because differences in cellular growth rate resulting from the G + C content of expressed GFP genes were explicit (Fig. 1B).

**Figure 2:**
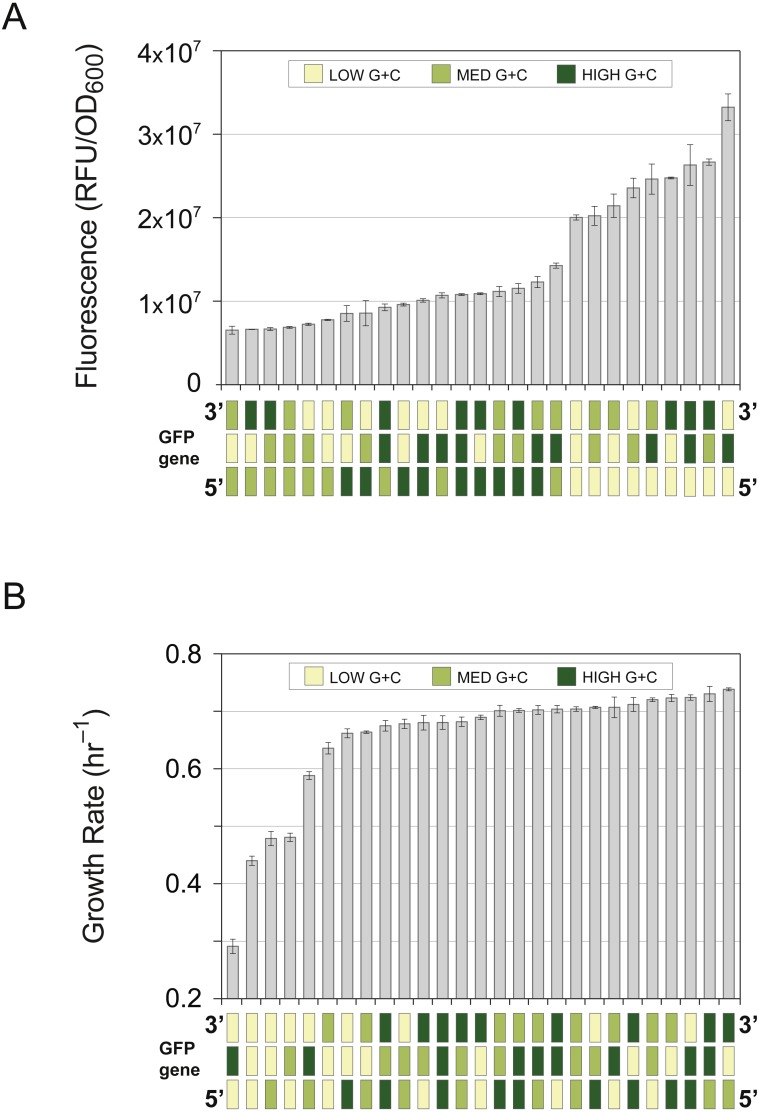
Gene expression and cellular growth rate of compositional mosaics. Recoded GFP genes (as shown in Fig. 1) were each subdivided into three fragments and shuffled to create all 27 possible full-length gene combinations. Each of the shuffled GFP genes encodes the identical protein sequence, and all constructs were cloned and expressed in the pFAB3857 vector. Gene diagrams on the *x*-axis show the mosaic structure of each of the tested genes, shaded according to the GC-content of each fragment: yellow; 41% G + C; light green; 50% G + C; dark green; 59% G + C. (A) Gene expression levels of mosaics GFP genes determined by intensity of cell fluorescence, with constructs arranged in order of increasing expression. Bars represent the mean ± standard deviation of three biological replicates. (B) Growth rates of *E. coli* strains expressing mosaic GFP genes. Fitness measurements the 27 GFP variants determined by exponential-phase growth rate, with constructs arranged in order of increasing growth rates. Bars represent the mean ± standard deviation of three biological replicates.

The 27 compositional mosaics in this library differed by up to 5-fold in GFP fluorescence (Fig. 2A). There was a negative association between fluorescence and overall genic G + C content (*r*^2^ = 0.141, *p* = 0.0006) as well as with overall genic CAI (*r*^2^ = 0.378, *p* < 0.0001) (Fig. S2). However, these trends were due, in large part, to the genes containing a L-fragment in the 5′-distal position, which together form a separate group (Fig. S3) independent of overall genic G + C content or overall genic CAI (Fig. S4). The genes with the 5′-distal L fragments displayed significantly higher levels of fluorescence than those with the 5′-distal M fragment (*p* < 0.0001) or the 5′-distal H fragment (*p* < 0.0001) (Fig. S5). These results indicate that some property associated with the G + C content of the 5′-distal fragment modulates expression levels. This finding aligns with prior studies of the translation initiation region that have found that low G + C sequences near the 5′-end tend to increase expression levels (Kudla et al., 2009; Goodman, Church & Kosuri, 2013). However, there has been some disagreement as to the underlying mechanism. The leading hypothesis is that lower levels of G + C content in the initiation region encourage ribosome loading due to a reduction in the strength of mRNA secondary structures (Kudla et al., 2009; Goodman, Church & Kosuri, 2013). However, it has also been argued that the effect is attributable to the low CAI values associated with many low G + C codons. Under this model, slower translation at the start of the transcript, as caused by low CAI codons, reduces ribosome collisions that are detrimental to expression (Tuller et al., 2010).

We observe a slightly stronger correlation between fluorescence and the G + C content of the 5′-distal fragment (*r*^2^ = 0.6219, *p* < 0.0001) than with CAI (*r*^2^ = 0.4793, *p* < 0.0001) (Fig. S6). It is curious that our constructs displayed this effect since all constructs were identical for the first 51 bp—a length thought to mitigate any sequence-specific effects caused by this region, as had been shown for native *E. coli* gene sequences (Goodman, Church & Kosuri, 2013). Additionally, we employed a bicistronic expression system, which has been shown to normalize translation initiation regardless of gene sequence (Mutalik et al., 2013). These results suggest that the size of the region affecting the efficiency of translation initiation is longer than previously established and that its effect cannot be wholly eliminated with the bicistronic expression format. In support of this view, another recent study has also shown that a more significant portion of the coding sequence may play a role in defining rates of translation initiation (Burkhardt et al., 2017).

**Figure 3:**
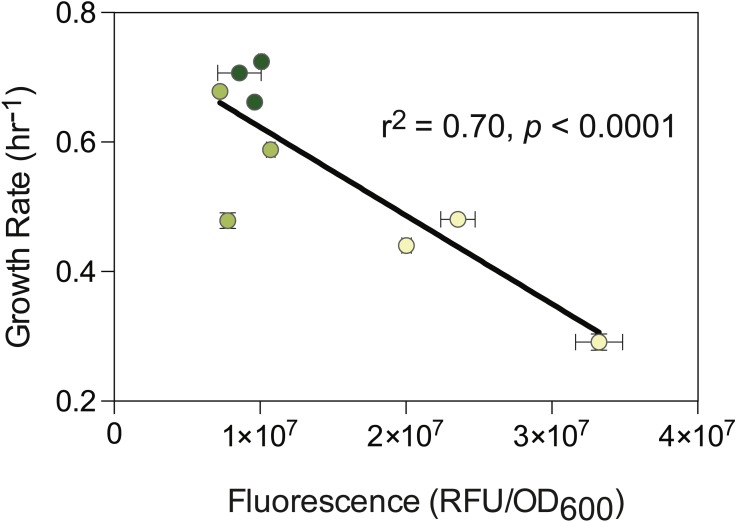
Association between expression level and cellular growth rates in *E. coli* harboring mosaic GFP genes that each contain the L-fragment in the terminal position. Shading denotes the GC-content of the 5′-distal fragment: yellow; 41% G + C; light green; 50% G + C; dark green; 59% G + C. Points represent the mean ± standard deviation of three biological replicates.

When assayed for their effects on cellular fitness, most of the 27 compositional mosaics resulted in only minor reductions in growth rates; however, those displaying severely reduced growth rates all contained an L-fragment in the terminal position (Fig. 2B). The magnitude of the fitness defect caused by the terminal L-fragment was associated with expression level: those strains expressing higher levels of the GFP gene, largely due to the G + C content of the 5′-distal fragment, displayed greater degrees of growth rate inhibition (*r*^2^ = 0.70, *p* < 0.0001) (Fig. 3).

### Sequence features causing cellular toxicity

Having established that the G + C content of the terminal-fragment of the GFP gene produces the greatest effect on cellular fitness, we sought to determine whether the overall G + C content of this region or, alternatively, if some specific sequence feature within this region dictated this effect. To distinguish between these hypotheses, we created two additional sets of terminal fragments:

In the first, we created fragments of the same overall G + C contents as the “canonical” L-fragment (≈41%) but recoded to contain a biased nucleotide content in the mRNA coding strand. Two such constructs were created: one in which the coding strand was enriched for cytosine and adenine bases (‘CA↑’) and another in which the coding strand was enriched for guanine and thymine bases (‘GT↑’). Importantly, these nucleotide-enriched constructs were not normalized for CAI values, and as a result, the CA↑ terminal fragment had a lower CAI value than the canonical-L and GT↑ terminal fragments (Table S1). This difference was largely driven by the presence of the rare CTA codon—the leucine codon most enriched in C and A—at all six leucine positions within the region.

The second set consisted of chimeric terminal fragments that were designed to dissect the precise location of any discrete sequence motif(s) responsible for the fitness detriment associated with a terminal L-fragment. Two chimeric terminal fragments were created, one containing half of the canonical L sequence followed by half of the canonical H-sequence (‘L/H’), and another containing half of the canonical H sequence followed by half of the canonical L sequence (‘H/L’). Both of these chimeras were assembled with the canonical low G + C L-5′-distal and L-proximal fragments to create full-length genes, and assayed in the pFAB3857 expression vector.

**Figure 4:**
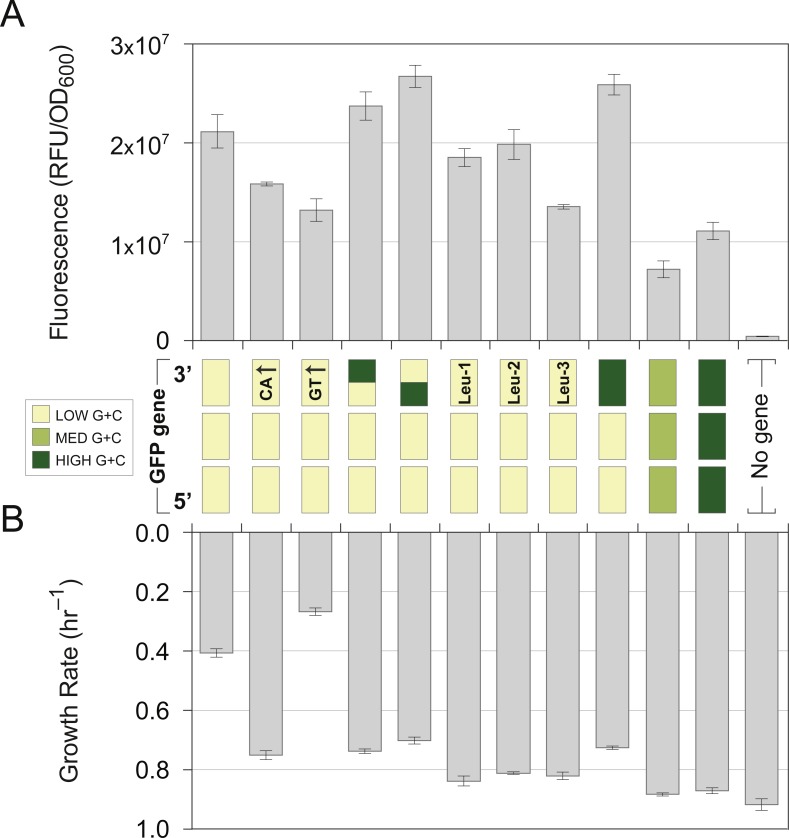
Synonymous nucleotide substitutions in the GFP terminal-fragment influence cellular growth rates and gene expression levels. Terminal-fragments of GFP genes (nucleotide positions 460–717) were modified to contain different sequence features, all of which preserved the GFP protein sequence, and assembled into full-length genes along with canonical low (yellow; 41% G + C) 5′-distal and proximal fragments. All constructs were cloned and expressed in plasmid pFAB3857. Notation of terminal-fragments is as follows: yellow unlabeled, the canonical low terminal-fragment of 41% G + C; CA↑, a low G + C fragment enriched in cytosine and adenine nucleotides in the coding strand; GT↑, a low G + C fragment enriched guanine and thymidine nucleotides in the coding strand; yellow-green chimera, a sequence that contains half of the canonical low G + C sequence followed half of the canonical high G + C sequence; green-yellow chimera, a sequence that contains half of the canonical high G + C sequence followed half of the canonical low G + C sequence; Leu-1, the canonical low G + C terminal-fragment possessing a synonymous mutation that altered the leucine codon at amino acid position 178 from CTG to CTA; Leu-2, the canonical low G + C terminal-fragment possessing synonymous mutations that altered the leucine codons at amino acid positions 194 and 195 from CTG to CTA; Leu-3, the canonical low G + C terminal-fragment possessing synonymous mutations that altered the leucine codons at amino acid positions 220 and 221 from CTG to CTA; dark green, the canonical high G + C terminal-fragment. Corresponding values for strains harboring full-length GFP-M (light green) or GFP-H (dark green) genes as well as a no-gene control are included for comparison. (A) Gene expression, as measured by cellular fluorescence intensity, of GFP variants and control strains. (B) Exponential-phase growth rates of GFP variants and control strains. Bars represent the mean ± standard deviation of three biological replicates.

The terminal gene-fragment variants in the first set (‘CA↑’ and ‘GT↑’) affected cellular fitness in different ways: although both constructs had the same overall base composition, the CA↑ fragment did not produce the fitness defect normally associated with the canonical L fragment. In contrast, the GT↑ terminal-fragment induced growth defects greater than that produced by the canonical L-fragment (Fig. 4). Note that both variants resulted in lower levels of GFP fluorescence relative to the canonical construct but that only the GT↑ fragment was associated with the fitness defect (Fig. 4).

The chimeric L/H and H/L variants both displayed about the same growth rate and expression level as the variant with canonical H-sequence throughout the terminal fragment (Fig. 4). These two sets of constructs, one of which altered base composition but not the overall %G + C of the terminal fragment and the other which swapped the positions of the GC-rich region in the terminal fragment, indicated that the toxicity associated with the canonical L-terminal fragment is not simply a function of %G + C but is mediated by a specific combination of sequence features within the mRNA.

### Attenuation of toxicity by rare codons

The toxicity of the canonical GFP-L gene variant made it impossible to recover clones when expressed by the three strongest promoters (Fig. 1B). Fortuitously, in an attempt to clone this fragment into the highly-expressing pFAB3665 vector, a single viable and fluorescent colony was recovered. This clone possessed a point mutation within the terminal-fragment of the GFP-L gene, converting G to A at nucleotide position 534. This mutation resulted in a synonymous substitution in the leucine codon at amino acid position 178, changing it from the preferred CTG codon to the rarely used CTA codon (Ikemura, 1985; Sharp & Li, 1987). This mutation, which restored cellular growth, was especially surprising given that it reduced the G + C content of the gene.

To characterize the effects of this mutation on cellular fitness and fluorescence properties, we cloned the GFP-L L178L sequence variant (Leu-1) into a lower-expressing pFAB3857 vector, which is viable and allows comparisons with the canonical GFP-L sequence. This L178L sequence variant exhibited growth rates similar to those expressing the GFP-M or H genes and near wild-type levels of fluorescence (Fig. 4). Therefore, this single synonymous mutation, which imparts a *lower* G + C content, suppresses the toxicity normally associated with the L-terminal fragment, but it does not operate through the reduction of gene expression.

This result led us to wonder if the suppression induced by the L178L mutation was due to a reduced rate of translation, as can occur at rare codons (Sørensen, Kurland & Pedersen, 1989; Gardin et al., 2014). Such attenuation sites have previously been shown to be involved in the co-translational folding of nascent proteins (Kimchi-Sarfaty et al., 2007; Komar, 2009; Zhang, Hubalewska & Ignatova, 2009) and may affect the fidelity of translation (Kramer & Farabaugh, 2007), either of which could have consequences for cellular fitness (Drummond & Wilke, 2009). To test if the L178L mutation is associated with translational attenuation—and if the specific location of this putative attenuation site is critical for suppression—we designed two variants of the canonical GFP-L terminal sequence in which the CTA leucine codon was substituted at various sites downstream of leucine 178. In one construct (Leu-2), we converted the CTG leucine codons at sites 194 and 195 to rare leucine CTA codons, and in the other (Leu-3), we converted the CTG leucine codons at sites 220 and 221 to CTA codons. These new terminal-fragments were assembled with the canonical low G + C L-5′-distal and L-proximal fragments to create full-length genes, and cloned into the pFAB3857 expression vector.

Each of the three rare-codon variants was able to rescue the fitness defect previously observed in the canonical L terminal-fragment, and like the original L178L mutation, each displayed only a minimal reduction in GFP fluorescence (Fig. 4). These results strengthen the hypothesis that the toxic effect of the canonical L terminal-fragment is due to a too-rapid rate of translation. Furthermore, each of these rare-codon constructs suppresses translational toxicity to a similar degree although they spanned a region of 42 amino acids.

### Reductions in global translation rates can alleviate cellular toxicity

Our finding that insertion of rare codons into the L terminal-fragment was sufficient to ameliorate the fitness defect associated with its expression suggested that the mechanism of suppression involved a reduction in translational rate through this region. It has previously been observed that eukaryotic proteins (such as GFP) are prone to misfolding when expressed in bacteria (Fukuda, Arai & Kuwajima, 2000; Chang et al., 2005). This propensity for misfolding has been attributed, in part, to the faster overall rate of translation elongation in bacteria, which can interfere with proper co-translational protein folding (Siller et al., 2010). We reasoned that if this faster rate of translation was responsible for the fitness defect observed from expression of GFP genes containing the L terminal-fragment then reducing the overall rate of translation in this context would have an alleviating effect on fitness. For these experiments, we took advantage of the fact that the mutations in the *rpsL* 30S ribosomal subunit S12 gene that confer resistance to the antibiotic streptomycin (Sm^R^) do so, in part, by reducing the overall rate of translation elongation (Kurland, Hughes & Ehrenberg, 1996).

**Figure 5:**
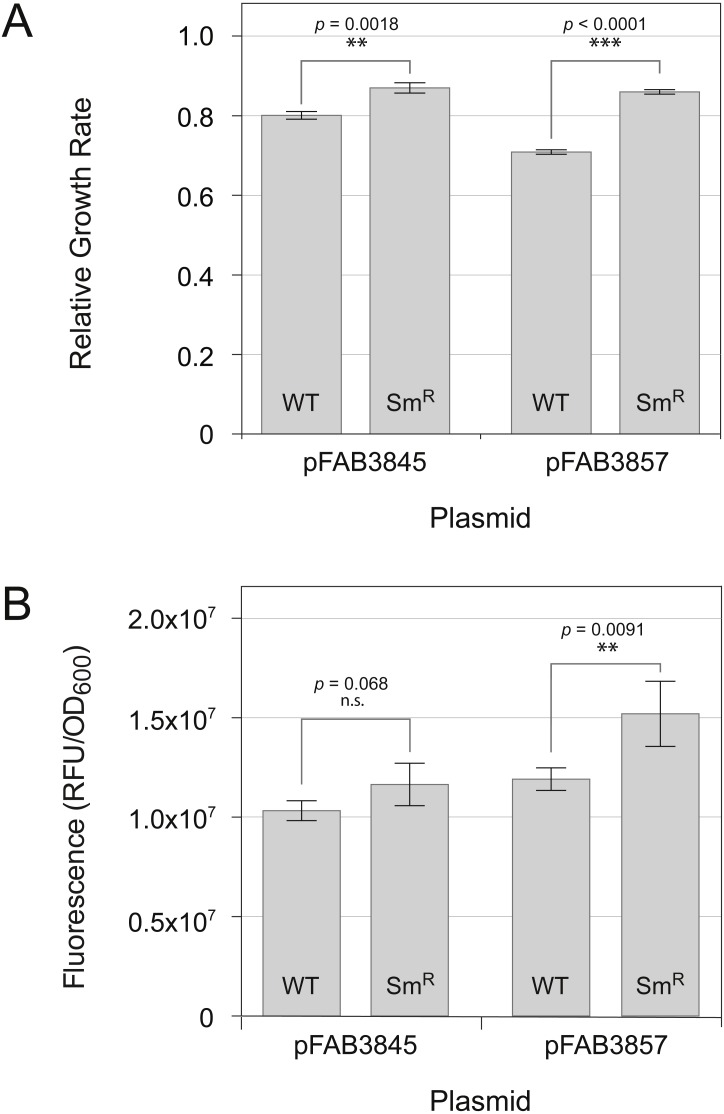
Effect of reduced translation rate on cellular fitness and gene expression. The canonical GFP-L gene, which possesses a consistently low G + C content over the entire gene sequence, was cloned and expressed in plasmid vectors with either a weak (pFAB3845) or strong (pFAB3857) promoter. Cloned constructs were assayed in the wild-type *E. coli* (WT, strain BW25113) and in a streptomycin resistant derivative (Sm^R^) whose global translation rate was reduced by introducing the P90Q mutation into the *rpsL* gene. (A) Comparison of relative growth rates of the WT and Sm^R^ strains expressing the canonical GFP-L gene from strong and weak promoters. Relative growth rates calculated as the average growth rate of GFP-expressing strain divided by the average growth rate of a no-expression control. (B) Comparison of gene expression levels in the WT and Sm^R^ strains expressing the canonical GFP-L gene from weak and strong promoters. Gene expression levels determined by measuring cellular fluorescence intensity. Results represent mean ± standard deviation of three biological replicates.

We introduced the P90Q mutation into the chromosomal *rpsL* sequence, producing a Sm^R^ strain with an error-restrictive phenotype (Holberger & Hayes, 2009) associated with reduced elongation rates and hyper-accurate translation (Kurland, Hughes & Ehrenberg, 1996). We then compared growth rates and fluorescence properties associated with the canonical GFP-L gene in wild-type and isogenic Sm^R^ strains using vectors of different expression strengths (pFAB3845 and pFAB3857). With both vectors, levels of growth rate inhibition were significantly lower in the Sm^R^ strain than in the wild-type strain (*p* = 0.0018 for pFAB3845; *p* < 0.0001 for pFAB3857, unpaired Student’s *t*-test), supporting the hypothesis that translation rates underlie the fitness defect associated with canonical L terminal-fragment (Fig. 5A). The level of GFP fluorescence was not found to be significantly different between both strains when the GFP-L gene was expressed from the pFAB3845 vector (*p* = 0.068, unpaired Student’s *t*-test), but was higher for the Sm^R^ strain when GFP-L was expressed from the pFAB3857 vector (*p* = 0.0091, unpaired Student’s *t*-test) (Fig. 5B). This result rules out the possibility that the growth rate enhancement of the Sm^R^ strains is a consequence of reduced protein expression. Taken together, these results suggest that expression of the GFP-L gene is toxic to wild-type cells because of a fitness cost associated with rapid translation.

## Discussion

We show that the fitness costs associated with the expression of GFP sequences of low G + C content are due almost entirely to the sequence within a very limited region near the 3′ terminus of the gene, indicating that selection does not act uniformly on base composition across the entire gene sequence. This finding indicates that the GC-effect on fitness is not caused by collective differences in the rate of translation at G/C *vs.* A/T sites but instead results from local sequence features that affect translation. Systematic dissection of the region associated with the fitness decrement revealed that introduction of either a high G + C sequence or a small number of rare synonymous codons were sufficient to restore fitness to normal levels, suggesting that both affected the same process.

A feature common to these alterations is that both the presence of certain rare codons and increases to the G + C content of mRNA can reduce rates of translation. Rare synonymous codons, like those used in our experiments, decrease rates of translation in *E. coli* on account of the limited concentrations of their isoaccepting tRNAs (Pedersen, 1984; Varenne et al., 1984; Sørensen, Kurland & Pedersen, 1989; Dong, Nilsson & Kurland, 1996; Chaney & Clark, 2015). Similarly, higher G + C contents tend to increase the stability of mRNA secondary structures, which can impede ribosome progression along the transcript (Somogyi et al., 1993; Wen et al., 2008; Chen et al., 2013). To confirm that translation speed is responsible for the observed fitness differences, we determined that expression of low G + C GFP variants are less deleterious when global rates of translation are reduced. Collectively, these findings all indicate that the primary source of the fitness defect caused by low G + C contents results from a regional rate of translation that is too fast.

Because the GFP gene variants differed in their overall base compositions, we attempted to control for differences in translation rate that might arise from this compositional variation by designing constructs with similar overall levels of adaptive codon usage bias. However, our results demonstrate that the G + C context in which a particular codon exists can alter the rate of translation in a way that impacts cellular fitness, even in genes, such as GFP, that have no functional relevance in *E. coli*. Such factors are not considered in current measures of codon adaptation, which are typically based solely on the frequency of each codon in the most highly expressed genes in a genome (Sharp & Li, 1987) or on the relative concentrations of isoaccepting tRNAs (Dos Reis, Savva & Wernisch, 2004). As Gorochowski et al. (2015) suggest, the failure to account for G + C content and its effect on the mRNA structural context of a given codon may be one reason why ribosomal profiling often fails to confirm the expected correlation between measures of codon adaptation and translational rate (Li, Oh & Weissman, 2012; Qian et al., 2012; Charneski & Hurst, 2013; Pop et al., 2014; Gorochowski et al., 2015).

That slowing the rate of translation imparts fitness benefits seems to contradict the view that a fast translation rate is generally beneficial because its ensures that the concentration of ribosomes does not becomes limiting during periods of rapid growth (Andersson & Kurland, 1990; Plotkin & Kudla, 2011). However, a fast rate of translation is not always ideal. For example, since proteins begin to fold as they emerge from the ribosome, the rate at which the sequence is translated will affect the conformational space that the protein can explore as it folds (Tsai et al., 2008; Komar, 2009; O’Brien et al., 2014). Attenuation sites often serve to slow or pause translation so that certain portions of the protein can adopt a particular configuration, and the absence of such sites can cause protein misfolding and result in altered functionality (Kimchi-Sarfaty et al., 2007) or loss of solubility and aggregation (Zhang, Hubalewska & Ignatova, 2009). The production of misfolded proteins is energetically costly and can cause toxic effects due to their altered structures (Bucciantini et al., 2002; Drummond & Wilke, 2008; Drummond & Wilke, 2009). And aside from directly affecting protein folding, a fast rate of translation can also negatively impact the fidelity of translation (Tubulekas & Hughes, 1993; Johansson, Zhang & Ehrenberg, 2012; Rodnina, 2012; Yang, Chen & Zhang, 2014) resulting in mutated or nonfunctional proteins that affect fitness (Drummond & Wilke, 2009). Additionally, this process explains why we observe higher fitness levels when low G + C variants were expressed in a Sm^R^ strain with hyper-accurate translation. Future experiments that directly measure rates of translation elongation and levels of misfolded or mistranslated proteins in relation to genic G + C content would strengthen support for these hypotheses.

If local levels of G + C content are under selection for the translational tuning of each protein, we might expect heterogeneity in the base composition of genes within a genome. But despite their differences in amino acid composition, the majority of genes within a bacterial genome are of similar G + C contents (Sueoka, 1962; Muto & Osawa, 1987; Karlin, Campbell & Mrázek, 1998), indicating that other factors that play a role in shaping base composition. For instance, translation rates can also be modulated by codon and amino acid usage (Ingolia et al., 2009; Pavlov et al., 2009; Gingold & Pilpel, 2011; Charneski & Hurst, 2013), the presence of anti-Shine-Dalgarno sequences (Li, Oh & Weissman, 2012; Fluman et al., 2014; Vasquez et al., 2016), and interactions between the mRNA transcript and other RNAs in the cell (Umu et al., 2016). The combined influence of these factors makes selection on base composition highly dependent on sequence context, potentially having a homogenizing effect on overall base composition.

Although low GC-variants of GFP cause fitness defects, it is not known where the GFP protein falls on the spectrum of protein-folding robustness in relation to its base composition. Because GFP is known to have some co-translational folding requirements (Kelkar et al., 2012) and originates from an eukaryote, which have markedly slower translation rates than bacteria (Siller et al., 2010), it may be prone to misfolding when translated at speeds typical of *E. coli*. Furthermore, the GFP protein is comprised mainly of ß-strands, which are thought to favor slower rates of translation, as evidenced by the preferential usage of poorly adapted codons in their coding sequences (Thanaraj & Argos, 1996). Assaying genes that are native to *E. coli* will help determine if resident sequences experience patterns of selection for base composition similar to those observed for GFP.

Horizontally acquired coding sequences are often of lower G + C content than genes native to a genome (Lawrence & Ochman, 1997; Daubin, Lerat & Perrière, 2003). In enteric bacteria, these acquired sequences are silenced by the H-NS protein, which targets and prevents the expression of sequences with low G + C contents (Lucchini et al., 2006; Navarre et al., 2006). The silencing of foreign genes has conventionally been viewed as a way to counteract the costs associated with the unregulated expression of superfluous genes. We have shown that low G + C sequences are deleterious at the level of translation, thereby providing an additional reason why such sequences need to be suppressed by H-NS.

The extent to which selection on genic G + C contents operates in bacteria other than *E. coli* is largely unknown. It might be expected that selection would be strongest in genomes with extreme GC-contents; however, the effect was observed in only one of the two high G + C organisms tested (Kelkar, Phillips & Ochman, 2015). Despite selection for higher GC-contents, bacterial species display base compositions as low as 13% G + C (McCutcheon & Moran, 2010). This variation in base composition among species could be ascribed to differences in the efficacy of selection caused by population-level processes or to other mechanisms that can compensate for detriments arising from suboptimal rates of translation. It is notable that endosymbiotic bacteria, which possess the most AT-biased genomes, typically overproduce the chaperone GroEL (Dale & Moran, 2006). This chaperone assists in folding the highly degraded protein sequences that accumulate in these genomes (Fares et al., 2002). Selection for higher GC-contents is likely to be too weak in these genomes to counteract the fixation of deleterious AT-mutations by drift, such that GroEL might also have a role in stabilizing proteins that fold incorrectly due to an improper rate of translation.

## Conclusions

The maintenance of a translation rate conducive to the production of properly folded proteins places selective pressure both on base composition and on codon usage, which both interact to determine local rates of translation. Since small changes in base composition can alter the rate at which key codons are decoded, it implies that selection can act on individual mutations that affect base composition. Thus, protein folding requirements for translational speed can serve a role in shaping base composition.

##  Supplemental Information

10.7717/peerj.4286/supp-1 Table S1 G + C contents, codon adaptation index (CAI) values, and mRNA folding energies of full-length GFP genes and sequence fragments L, M, and H denote Low, Med, and High G + C GFP sequence fragments as described in main text. See Table S2 for nucleotide sequences.

10.7717/peerj.4286/supp-2 Table S2 Nucleotide sequences of GFP sequence fragments

10.7717/peerj.4286/supp-3 Figure S1 Association between GFP expression levels and growth rates of strains included in Fig. 1 GFP genes were recoded at synonymous sites to have consistently low (41%), medium (50%) or high (59%) G + C content over the entire gene. Recoded GFP genes were expressed in pFAB vectors containing promoters of varying strength. Expression levels determined by intensity of cell fluorescence. Bars represent the mean ± standard deviation of three biological replicates.

10.7717/peerj.4286/supp-4 Figure S2 Association between GFP expression levels (as measured by cell fluorescence) and overall GC-content (A) or overall CAI (B) for the mosaic GFP genes Points represent the mean ± standard deviation of three biological replicates.

10.7717/peerj.4286/supp-5 Figure S3 Association between GFP expression levels (as measured by cell fluorescence) and overall GC-content (A) or overall CAI (B) for the mosaic GFP genes Points are colored according to the GC-content of the 5’-distal fragment as follows: yellow; 43% G + C; light green; 53% G + C; dark green; 61% G + C. Points represent the mean ± standard deviation of three biological replicates.

10.7717/peerj.4286/supp-6 Figure S4 Comparison of overall genic GC-content (A) and overall genic CAI (B) with the GC-content of the 5’-distal fragment (L, 43% G + C; M, 53% G + C; H, 61% G + C) of the mosaic GFP genes **p < 0.001, ***p < 0.0001 (Mann–Whitney–Wilcoxon test).

10.7717/peerj.4286/supp-7 Figure S5 Mosaic GFP genes containing the 5’-distal L fragment express at higher levels than compositional mosaics containing M or H 5’-distal fragments Expression levels determined by intensity of cell fluorescence. GC-contents of the 5’-distal fragment are as follows: L, 43% G + C; M, 53% G + C; H, 61% G + C). ***p < 0.0001 (Mann–Whitney–Wilcoxon test).

10.7717/peerj.4286/supp-8 Figure S6 Association between GFP expression levels (as measured by cell fluorescence) and the GC-content (A) or CAI (B) of the 5’-distal fragment of mosaic GFP genes

10.7717/peerj.4286/supp-9 Data S1 Raw data 9 raw datasets associated with figures in the text.

## Additional Information and Declarations

### Data Availability

The following information was supplied regarding data availability: The raw data has been provided as Supplemental File.
