# Supplementary material for: Local genic base composition impacts protein production and cellular fitness
Source: PeerJ. 2018 Jan 16;6:e4286. doi: 10.7717/peerj.4286 (PMC5774297; doi:10.7717/peerj.4286)
Supplement: Table S1 — L, M, and H denote Low, Med, and High G + C GFP sequence fragments as described in main text. See Table S2 for nucleotide sequences. [file peerj-06-4286-s001.docx]

**Table S1**: G+C contents, codon adaptation index (CAI) values (1), and mRNA folding energies of full-length GFP genes and sequence fragments. L, M, and H denote Low, Med, and High G+C GFP sequence fragments as described in main text. See **Table S2** for nucleotide sequences.

| **Sequence** | **%G+C** | **CAI** | **Folding Energy RNALfold**(2)  **(kcal mol^-1^)** |
| --- | --- | --- | --- |
| **Full-Length Genes:** |  |  |  |
| GFP-L | 41.28 | 0.589 | -158.2 |
| GFP-M | 50.77 | 0.607 | -199.2 |
| GFP-H | 59.27 | 0.586 | -213.1 |
| **5' Distal Fragments:** |  |  |  |
| L | 42.75 | 0.566 | -52.8 |
| M | 52.94 | 0.730 | -69.0 |
| H | 60.78 | 0.601 | -83.8 |
| **Proximal Fragments:** |  |  |  |
| L | 40.20 | 0.637 | -42.8 |
| M | 51.96 | 0.616 | -49.6 |
| H | 57.84 | 0.618 | -50.2 |
| **Terminal Fragments:** |  |  |  |
| L | 40.70 | 0.576 | -58.0 |
| M | 47.67 | 0.501 | -71.1 |
| H | 58.91 | 0.547 | -71.0 |
| **Terminal Fragment Variants:** |  |  |  |
| L-CA↑ | 39.53 | 0.212 | -32.2 |
| L-GT↑ | 41.09 | 0.488 | -69.5 |
| H/L | 50.78 | 0.580 | -69.5 |
| L/H | 48.84 | 0.543 | -57.9 |
| L-Leu-1 (L178L) | 40.31 | 0.537 | -58.0 |
| L-Leu-2 (L194L, L195L) | 39.92 | 0.501 | -55.4 |
| L-Leu-3 (L220L, L221L) | 39.92 | 0.501 | -56.6 |
| **Compositional Mosaics (shown in 5’→3’ order):** |  |  |  |
| LLL | 41.28 | 0.589 | -185.2 |
| LLM | 43.79 | 0.560 | -172.0 |
| LLH | 47.84 | 0.578 | -173.0 |
| LML | 44.63 | 0.583 | -171.4 |
| LMM | 47.14 | 0.555 | -184.1 |
| LMH | 51.19 | 0.573 | -184.1 |
| LHL | 46.30 | 0.584 | -173.6 |
| LHM | 48.81 | 0.555 | -186.0 |
| LHH | 52.86 | 0.574 | -185.3 |
| MLL | 44.91 | 0.645 | -174.6 |
| MLM | 47.42 | 0.613 | -188.6 |
| MLH | 51.46 | 0.633 | -187.5 |
| MML | 48.26 | 0.639 | -184.0 |
| MMM | 50.77 | 0.607 | -199.2 |
| MMH | 54.81 | 0.627 | -198.8 |
| MHL | 49.93 | 0.639 | -184.0 |
| MHM | 52.44 | 0.608 | -199.1 |
| MHH | 56.49 | 0.628 | -198.3 |
| HLL | 47.70 | 0.602 | -186.8 |
| HLM | 50.21 | 0.572 | -201.0 |
| HLH | 54.25 | 0.591 | -200.3 |
| HML | 51.05 | 0.596 | -200.9 |
| HMM | 53.56 | 0.567 | -216.1 |
| HMH | 57.60 | 0.585 | -215.7 |
| HHL | 52.72 | 0.597 | -199.9 |
| HHM | 55.23 | 0.567 | -215.2 |
| HHH | 59.27 | 0.586 | -213.1 |
| LL(L-CA↑) | 40.86 | 0.411 | -132.4 |
| LL(L-GT↑) | 41.42 | 0.555 | -170.2 |
| LL(H/L) | 44.91 | 0.591 | -169.5 |
| LL(L/H) | 44.21 | 0.577 | -160.2 |
| LL(L-Leu-1) | 41.14 | 0.574 | -158.3 |
| LL(L-Leu-2) | 41.00 | 0.560 | -155.7 |
| LL(L-Leu-3) | 41.00 | 0.560 | -156.8 |

1. Sharp PM, Li WH (1987) The codon adaptation index-a measure of directional synonymous codon usage bias, and its potential applications. *Nucleic Acids Res* 15(3):1281–1295.

2. Hofacker IL, Priwitzer B, Stadler PF (2004) Prediction of locally stable RNA secondary structures for genome-wide surveys. *Bioinformatics* 20(2):186–190.
