# Supplementary material for: Local genic base composition impacts protein production and cellular fitness
Source: PeerJ. 2018 Jan 16;6:e4286. doi: 10.7717/peerj.4286 (PMC5774297; doi:10.7717/peerj.4286)
Supplement: Table S2 [file peerj-06-4286-s002.docx]

**Table S2:** Nucleotide sequences of GFP sequence fragments.

| **Name** | **Sequence (5’→3’)** |
| --- | --- |
| 5’ Distal-L | ATGCGTAAAGGCGAGGAGCTGTTCACTGGTGTCGTCCCTATTCTGGTGGAATTAGATGGCGATGTTAATGGCCATAAATTCAGTGTTCGTGGTGAAGGCGAAGGTGATGCAACTAATGGTAAACTGACTCTGAAATTTATTTGCACTACTGGTAAATTACCGGTTCCGTGGCCGACTTTAGTTACTACTCTGACCTATGGTGTTCAATGTTTTGCGCGTTATCCGGATCATATGAAACAACATGATTTCTTTAAG |
| Proximal-L | AGTGCGATGCCAGAAGGCTATGTGCAGGAACGTACCATCAGTTTCAAAGATGATGGTACTTACAAAACCCGCGCAGAAGTTAAATTTGAAGGCGATACTCTGGTTAATCGTATTGAACTGAAAGGCATTGATTTTAAAGAAGATGGCAATATCCTGGGTCACAAACTGGAATACAACTTTAATAGTCACAACGTTTACATCACC |
| Terminal-L | GCAGATAAACAGAAAAATGGTATTAAAGCAAATTTTAAAATTCGTCATAATGTTGAAGATGGTAGCGTTCAACTGGCGGATCATTATCAACAAAATACCCCGATTGGTGATGGTCCGGTTCTGCTGCCAGATAATCATTATCTGTCTACCCAAAGTGTTCTGAGCAAAGATCCGAATGAAAAACGTGATCATATGGTTCTGCTGGAGTTCGTAACCGCAGCGGGCATCACGCATGGTATGGATGAACTGTACAAATAA |
| 5’-Distal-M | ATGCGTAAAGGCGAGGAGCTGTTCACTGGTGTCGTCCCTATTCTGGTGGAACTGGATGGCGATGTGAATGGTCATAAGTTCTCTGTGCGTGGCGAAGGTGAAGGTGACGCTACCAACGGCAAACTGACCCTGAAGTTCATCTGCACCACTGGTAAACTGCCGGTTCCGTGGCCGACTCTGGTTACTACTCTGACTTATGGCGTGCAGTGCTTCGCTCGTTACCCGGACCACATGAAACAGCACGATTTCTTCAAG |
| Proximal-M | TCCGCGATGCCGGAAGGCTATGTGCAGGAGCGCACCATTTCTTTCAAAGACGACGGCACCTATAAAACCCGTGCGGAAGTGAAGTTCGAGGGCGATACCCTGGTTAATCGCATTGAGCTGAAAGGCATTGACTTTAAAGAGGACGGCAACATCCTGGGCCACAAGCTGGAGTACAACTTCAACAGCCACAACGTCTACATCACG |
| Terminal-M | GCCGACAAGCAGAAGAACGGCATCAAGGCGAATTTTAAAATCCGTCATAACGTTGAGGATGGCTCTGTGCAGCTTGCGGATCATTATCAACAAAATACCCCGATTGGTGATGGTCCGGTTCTGCTGCCAGATAATCATTATCTGAGCACCCAAAGCGTCCTTTCCAAAGATCCGAACGAGAAGCGCGACCATATGGTTCTTCTTGAGTTCGTAACCGCAGCGGGCATCACGCATGGTATGGATGAACTGTACAAATAA |
| 5’-Distal-H | ATGCGTAAAGGCGAGGAGCTGTTCACTGGTGTCGTCCCTATTCTGGTGGAACTGGATGGCGATGTCAACGGCCACAAGTTCAGCGTGCGCGGCGAGGGCGAAGGCGACGCCACCAACGGCAAACTGACCCTGAAGTTCATCTGTACCACCGGCAAACTGCCGGTCCCCTGGCCGACCCTGGTCACCACCCTGACCTACGGCGTCCAGTGCTTCGCCCGCTACCCGGACCACATGAAGCAGCATGACTTCTTCAAG |
| Proximal-H | TCCGCCATGCCGGAAGGCTACGTGCAGGAACGCACCATCAGCTTCAAGGACGACGGCACCTACAAAACCCGCGCGGAGGTGAAATTCGAGGGCGATACGCTGGTCAACCGCATCGAGCTGAAGGGCATCGACTTCAAGGAGGACGGCAACATCCTGGGCCACAAGCTGGAGTACAACTTCAACAGCCACAACGTCTACATCACG |
| Terminal-H | GCCGACAAGCAGAAGAACGGCATCAAGGCGAACTTCAAGATCCGCCACAACGTGGAGGACGGCAGCGTGCAGCTGGCCGACCACTACCAGCAAAATACCCCCATCGGCGACGGCCCCGTCCTGCTGCCCGACAACCACTACCTGAGCACCCAAAGCGTCCTGTCCAAAGACCCGAACGAGAAGCGCGACCACATGGTCCTGCTGGAGTTCGTAACCGCAGCGGGCATCACGCATGGTATGGATGAACTGTACAAATAA |
| Terminal-L-CA↑ | GCAGATAAACAAAAGAACGGAATAAAAGCAAACTTCAAAATAAGACATAACGTAGAAGATGGATCAGTACAACTAGCAGATCACTACCAACAAAACACACCAATAGGAGATGGACCAGTACTACTACCAGACAACCACTACCTATCAACACAAAGCGTACTATCAAAAGATCCAAATGAAAAAAGAGACCACATGGTACTACTAGAGTTCGTAACCGCAGCGGGCATCACGCATGGTATGGATGAACTGTACAAATAA |
| Terminal-L-GT↑ | GCTGATAAGCAGAAGAATGGTATTAAGGCTAATTTTAAGATTCGTCATAATGTTGAGGATGGTAGTGTTCAGTTGGCTGATCATTATCAGCAGAATACTCCTATTGGTGATGGTCCTGTTTTGCTGCCGGATAATCATTATTTGAGTACTCAGAGTGTTCTGTCTAAGGATCCTAATGAAAAGCGTGATCATATGGTTCTGCTGGAGTTCGTAACCGCAGCGGGCATCACGCATGGTATGGATGAACTGTACAAATAA |
| Terminal-L/H | GCAGATAAACAGAAAAATGGTATTAAAGCAAATTTTAAAATTCGTCATAATGTTGAAGATGGTAGCGTTCAACTGGCGGATCATTATCAACAAAATACCCCGATCGGCGACGGCCCCGTCCTGCTGCCCGACAACCACTACCTGAGCACCCAAAGCGTCCTGTCCAAAGACCCGAACGAGAAGCGCGACCACATGGTCCTGCTGGAGTTCGTAACCGCAGCGGGCATCACGCATGGTATGGATGAACTGTACAAATAA |
| Terminal-H/L | GCCGACAAGCAGAAGAACGGCATCAAGGCGAACTTCAAGATCCGCCACAACGTGGAGGACGGCAGCGTGCAGCTGGCCGACCACTACCAGCAAAATACCCCCATTGGTGATGGTCCGGTTCTGCTGCCAGATAATCATTATCTGTCTACCCAAAGTGTTCTGAGCAAAGATCCGAATGAAAAACGTGATCATATGGTTCTGCTGGAGTTCGTAACCGCAGCGGGCATCACGCATGGTATGGATGAACTGTACAAATAA |
| Terminal-L-Leu-1 | GCAGATAAACAGAAAAATGGTATTAAAGCAAATTTTAAAATTCGTCATAATGTTGAAGATGGTAGCGTTCAACTAGCGGATCATTATCAACAAAATACCCCGATTGGTGATGGTCCGGTTCTGCTGCCAGATAATCATTATCTGTCTACCCAAAGTGTTCTGAGCAAAGATCCGAATGAAAAACGTGATCATATGGTTCTGCTGGAGTTCGTAACCGCAGCGGGCATCACGCATGGTATGGATGAACTGTACAAATAA |
| Terminal-L-Leu-2 | GCAGATAAACAGAAAAATGGTATTAAAGCAAATTTTAAAATTCGTCATAATGTTGAAGATGGTAGCGTTCAACTGGCGGATCATTATCAACAAAATACCCCGATTGGTGATGGTCCGGTTCTACTACCAGATAATCATTATCTGTCTACCCAAAGTGTTCTGAGCAAAGATCCGAATGAAAAACGTGATCATATGGTTCTGCTGGAGTTCGTAACCGCAGCGGGCATCACGCATGGTATGGATGAACTGTACAAATAA |
| Terminal-L-Leu-3 | GCAGATAAACAGAAAAATGGTATTAAAGCAAATTTTAAAATTCGTCATAATGTTGAAGATGGTAGCGTTCAACTGGCGGATCATTATCAACAAAATACCCCGATTGGTGATGGTCCGGTTCTGCTGCCAGATAATCATTATCTGTCTACCCAAAGTGTTCTGAGCAAAGATCCGAATGAAAAACGTGATCATATGGTTCTACTAGAGTTCGTAACCGCAGCGGGCATCACGCATGGTATGGATGAACTGTACAAATAA |
