# Supplementary material for: Local genic base composition impacts protein production and cellular fitness
Source: PeerJ. 2018 Jan 16;6:e4286. doi: 10.7717/peerj.4286 (PMC5774297; doi:10.7717/peerj.4286)
Supplement: Figure S1 — GFP genes were recoded at synonymous sites to have consistently low (41%), medium (50%) or high (59%) G + C content over the entire gene. Recoded GFP genes were expressed in pFAB vectors containing promoters of varying strength. Expression levels determined by intensity of cell fluorescence. Bars represent the mean ± standard deviation of three biological replicates. [file peerj-06-4286-s003.pdf]

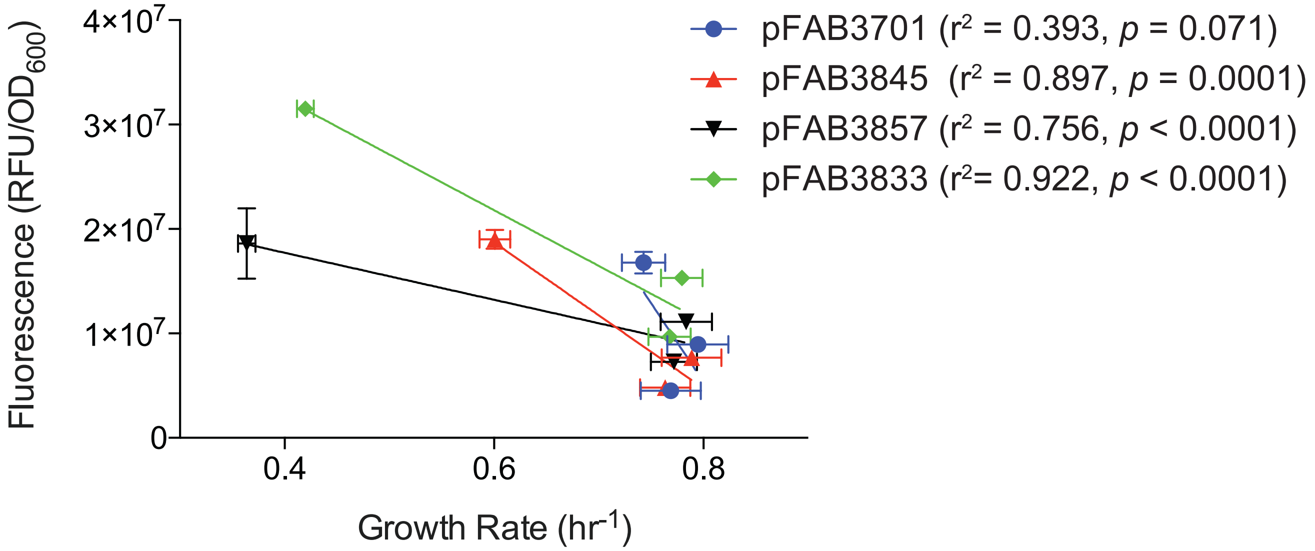

Figure S1: Association between GFP expression levels and growth rates of strains included in Figure 1. GFP genes were recoded at synonymous sites to have consistently low (41%), medium (50%) or high (59%) G+C content over the entire gene. Recoded GFP genes were expressed in pFAB vectors containing promoters of varying strength. Expression levels determined by intensity of cell fluorescence. Bars represent the mean ± standard deviation of three biological replicates.
