# Supplementary material for: Local genic base composition impacts protein production and cellular fitness
Source: PeerJ. 2018 Jan 16;6:e4286. doi: 10.7717/peerj.4286 (PMC5774297; doi:10.7717/peerj.4286)
Supplement: Figure S2 — Points represent the mean ± standard deviation of three biological replicates. [file peerj-06-4286-s004.pdf]

Figure S2

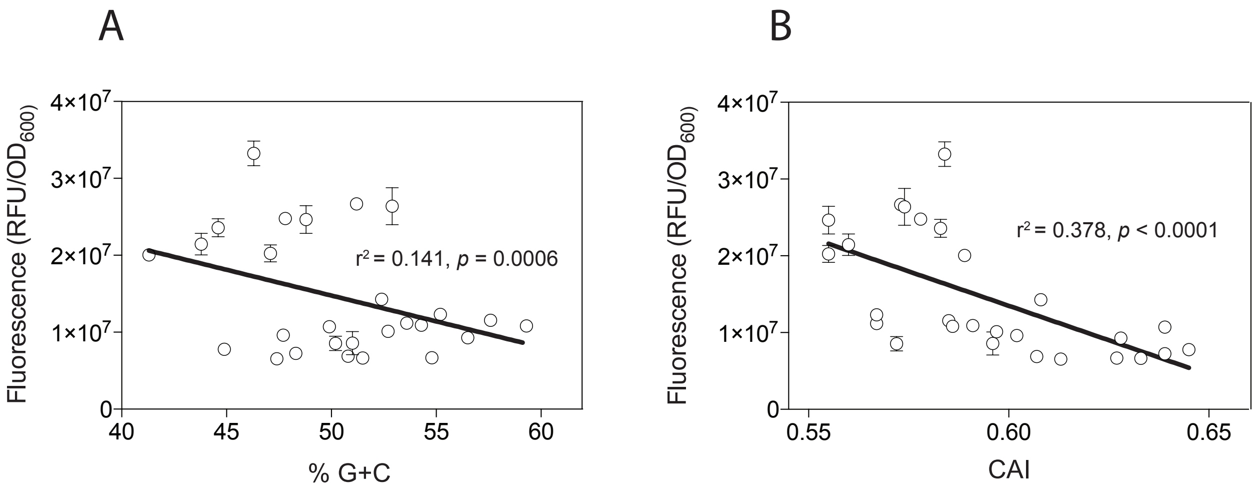

Figure S2: Association between GFP expression levels (as measured by cell fluorescence) and overall GC-content (**A**) or overall CAI (**B**) for the mosaic GFP genes. Points represent the mean ± standard deviation of three biological replicates.
