# Supplementary material for: Local genic base composition impacts protein production and cellular fitness
Source: PeerJ. 2018 Jan 16;6:e4286. doi: 10.7717/peerj.4286 (PMC5774297; doi:10.7717/peerj.4286)
Supplement: Figure S3 — Points are colored according to the GC-content of the 5’-distal fragment as follows: yellow; 43% G + C; light green; 53% G + C; dark green; 61% G + C. Points represent the mean ± standard deviation of three biological replicates. [file peerj-06-4286-s005.pdf]

Figure S3

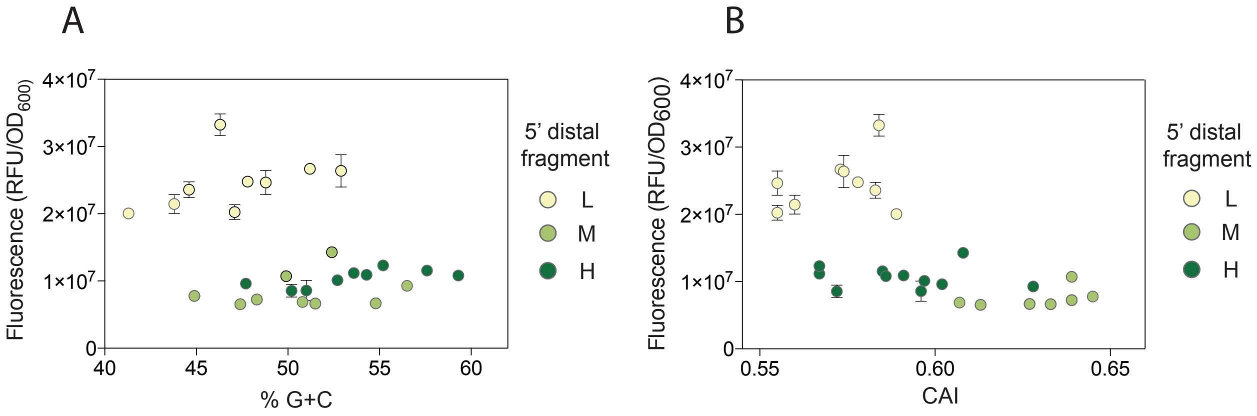

Figure S3: Association between GFP expression levels (as measured by cell fluorescence) and overall GC-content (**A**) or overall CAI (**B**) for the mosaic GFP genes. Points are colored according to the GC-content of the 5'-distal fragment as follows: yellow; 43% G+C; light green; 53% G+C; dark green; 61% G+C. Points represent the mean ± standard deviation of three biological replicates.
