# Supplementary material for: Local genic base composition impacts protein production and cellular fitness
Source: PeerJ. 2018 Jan 16;6:e4286. doi: 10.7717/peerj.4286 (PMC5774297; doi:10.7717/peerj.4286)
Supplement: Figure S4 — **p < 0.001, ***p < 0.0001 (Mann–Whitney–Wilcoxon test). [file peerj-06-4286-s006.pdf]

Figure S4

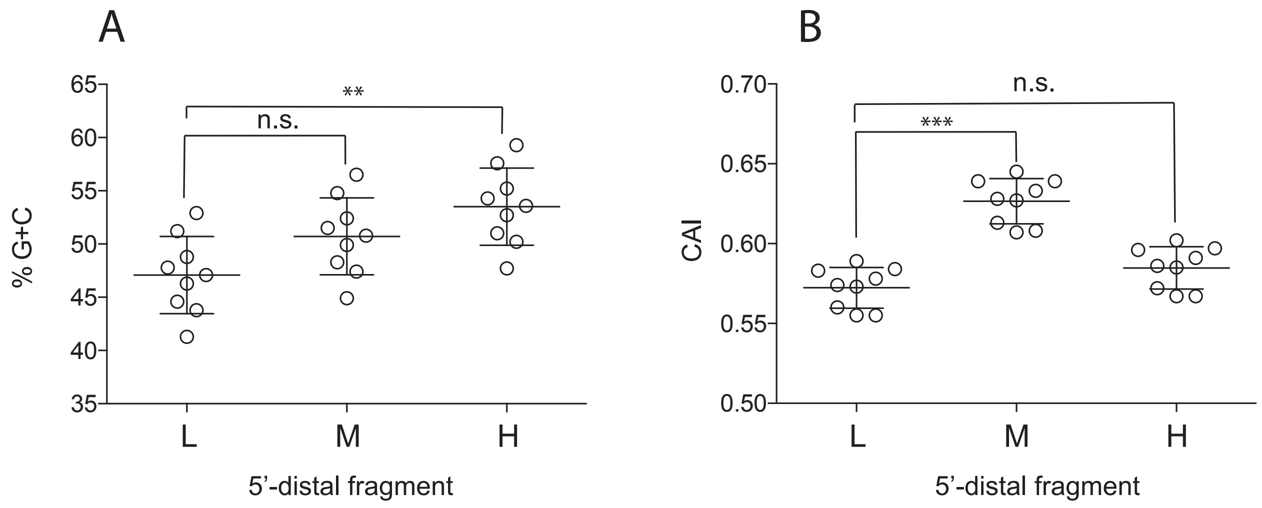

Figure S4: Comparison of overall genic GC-content (**A**) and overall genic CAI (**B**) with the GC-content of the 5’-distal fragment (L, 43% G+C; M, 53% G+C; H, 61% G+C) of the mosaic GFP genes. **$p < 0.001$, ***$p < 0.0001$ (Mann–Whitney–Wilcoxon test).
