# Supplementary material for: Local genic base composition impacts protein production and cellular fitness
Source: PeerJ. 2018 Jan 16;6:e4286. doi: 10.7717/peerj.4286 (PMC5774297; doi:10.7717/peerj.4286)
Supplement: Figure S5 — Expression levels determined by intensity of cell fluorescence. GC-contents of the 5’-distal fragment are as follows: L, 43% G + C; M, 53% G + C; H, 61% G + C). ***p < 0.0001 (Mann–Whitney–Wilcoxon test). [file peerj-06-4286-s007.pdf]

Figure S5

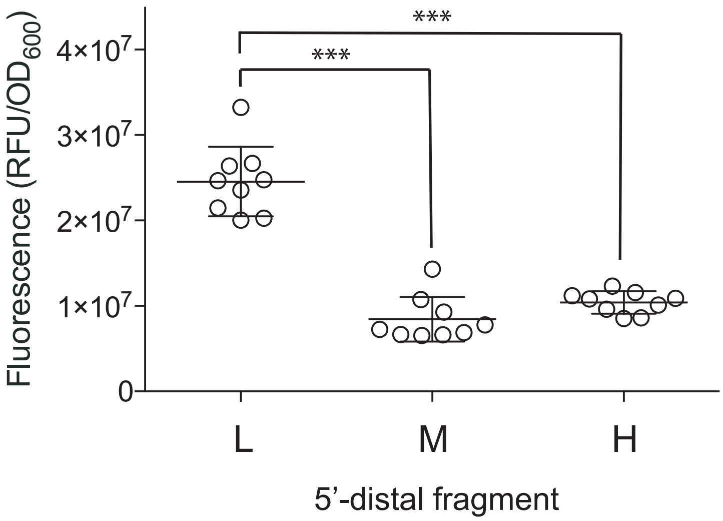

Figure S5: Mosaic GFP genes containing the 5'-distal L fragment express at higher levels than compositional mosaics containing M or H 5'-distal fragments. Expression levels determined by intensity of cell fluorescence. GC-contents of the 5'-distal fragment are as follows: L, 43% G+C; M, 53% G+C; H, 61% G+C). ***$p < 0.0001$ (Mann–Whitney–Wilcoxon test).
