# Supplementary material for: Local genic base composition impacts protein production and cellular fitness
Source: PeerJ. 2018 Jan 16;6:e4286. doi: 10.7717/peerj.4286 (PMC5774297; doi:10.7717/peerj.4286)
Supplement: Figure S6 [file peerj-06-4286-s008.pdf]

Figure S6

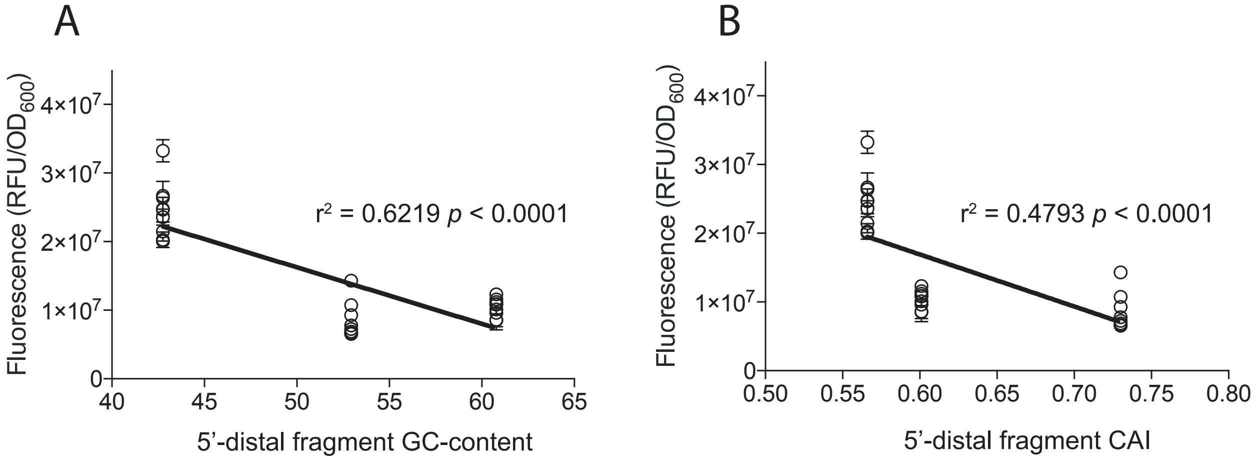

Figure S6: Association between GFP expression levels (as measured by cell fluorescence) and the GC-content (**A**) or CAI (**B**) of the 5'-distal fragment of mosaic GFP genes.
